# Vitamin D nutritional status and the influencing factors among children and adolescents

**DOI:** 10.3389/fpubh.2025.1553077

**Published:** 2025-06-11

**Authors:** Yan Zou, Li-chun Huang, Dong Zhao, Meng-jie He, Dan Han, Danting Su, Peiwei Xu, Ronghua Zhang

**Affiliations:** Zhejiang Provincial Center for Disease Control and Prevention, Hangzhou, China

**Keywords:** 25-(OH)D, vitamin D deficiency, outdoor activity, children, China

## Abstract

**Objective:**

To assess the nutritional status of vitamin D and to analyze the influencing factors of vitamin D deficiency among children and adolescents.

**Methods:**

Data from 1,827 children and adolescents aged 6–17 years from the nutrition and health surveillance of Zhejiang province, China, were analyzed. The serum concentration of 25-(OH)D were measured using High-Performance Liquid Chromatography-Tandem Mass Spectrometry. The prevalence of vitamin D deficiency and insufficiency were calculated. Ordinal regression were used to identify the influencing factors of vitamin D deficiency and insufficiency.

**Results:**

The mean 25-(OH)D concentration was 20.84 ± 6.34 μg/L. Among the participants, 37.4% had vitamin D insufficiency (25-(OH)D <20 μg/L) and 7.8% had vitamin D deficiency (25-(OH)D <12 μg/L). Age group (Wald *c*^2^ = 5.921, *p* = 0.015), sex (Wald *c*^2^ = 6.206, *p* = 0.013), overweight/obesity (Wald *c*^2^ = 3.894, *p* = 0.048), and outdoor activity time (Wald *c*^2^ = 4.113, *p* = 0.043) were the influencing factors of children and adolescents with insufficiency/deficiency vitamin D status.

**Conclusions:**

Our study assessed the nutritional status of vitamin D and analyzed the influencing factors of vitamin D deficiency among children and adolescents in the eastern coastal areas of China. The results indicated a significant prevalence of vitamin D insufficiency and deficiency. Key influencing factors included age group, sex, overweight/obesity, and outdoor activity time. These findings highlight the need for targeted interventions to improve vitamin D levels in children and adolescents. Specifically, promoting adequate outdoor activity and addressing overweight/obesity could be effective strategies to enhance vitamin D status in this population.

## Introduction

Vitamin D is a steroid hormone obtained through dietary intake and by endogenous synthesis requiring exposure to sunlight. Two forms of vitamin D are of practical importance: vitamin D2 (ergocalciferol—plant-derived) and vitamin D3 (cholecalciferol—animal-derived) (1). Serum 25(OH)D is the primary indicator of vitamin D status, which reflects the adequacy of circulating 25-(OH)D levels, and is the optimal method for assessing vitamin D status (2). Vitamin D plays a crucial role in the control of calcium and phosphorus metabolism, and is an essential determinant of bone health in childhood and adolescence (3). Clinical and experimental models suggest associations between Vitamin D and the gut microbiome, which may enhance anti-inflammatory effects and support metabolic health. These associations could potentially influence gut-related behavioral changes and diseases, although causality remains uncertain (4). Prospective studies reported moderate to strong inverse associations between 25(OH)D concentrations and cardiovascular diseases, serum lipid concentrations, inflammation, glucose metabolism disorders, weight gain, infectious diseases, multiple sclerosis, mood disorders, declining cognitive function, impaired physical functioning, and all-cause mortality (5, 6). There are variable definitions of vitamin D deficiency, based on different thresholds of serum 25-hydroxyvitamin D (25(OH)D) (7). No matter which standard is used, vitamin D deficiency is a serious public health problem, affecting nearly 50% of the global population (8, 9).

New life style habits, current “epidemics” of obesity in children and adolescents worldwide, and other preventable risk factors may play a role in favoring the occurrence of vitamin D deficiency. According to recent studies, the prevalence of obesity among children and adolescents in China has been increasing significantly. For example, the mean prevalence of obesity rose from 0.10% in 1985 to 8.25% in 2019 (10). This rising trend in obesity rates is a significant public health concern, as obesity is associated with multiple health risks, including vitamin D deficiency.

The importance of assessing vitamin D status specifically in children and adolescents is particularly significant. During childhood and adolescence, the body undergoes rapid growth and development, making these periods critical for establishing and maintaining optimal vitamin D levels (11). Vitamin D is essential for proper calcium absorption and bone mineralization. Vitamin D deficiency in early life has been linked to long-term health consequences, including increased risk of autoimmune diseases, cardiovascular diseases, and impaired cognitive development. Furthermore, emerging evidence suggests that vitamin D status may also influence mental health in children and adolescents. For instance, recent studies have reported associations between vitamin D deficiency and depressive symptoms (12). Therefore, assessing vitamin D status in children and adolescents is crucial for early identification and intervention to prevent both immediate and long-term health issues.

No provincial representative evidence on children and adolescents' vitamin D status in the eastern coastal areas of China. The present study aimed to learn the nutritional status of vitamin D and to analyze the influencing factors of vitamin D deficiency among children and adolescents.

## Methods

### Study design and participants

This provincial nutrition surveillance in 2022 is a population-based household survey including children and adolescents aged 6–17 years. Participants were divided into two main age groups: children (6–11 years) and adolescents (12–17 years). For detailed analysis, these groups were further subdivided into four age subgroups: 6–8, 9–11, 12–14, and 15–17 years. We calculated the sample size using the formula $\text{N }=\;\frac{{\text{deffu}}^{2}\text{P}\left(1-\text{P}\right)}{{\text{d}}^{2}}$. “deff” is the design efficiency, and in the sample size calculation of this study, the “deff” value is set to 1.5. “d” is the relative error , which is controlled at 15% in this study. The confidence level is set at 95% (bilateral). “P” is the prevalence rate of obesity rate of previous year. Obesity was chosen as the primary determinant for sample size calculation because it is a significant public health issue and is associated with multiple health risks. Additionally, obesity is a key indicator in our study, and ensuring adequate sample size for this parameter helps in accurately assessing its prevalence and impact on the study population. The study was conducted during the summer months (June to August 2022) to minimize potential seasonal variations in biomarkers. This period was chosen because it is known to have a significant impact on vitamin D levels due to increased sunlight exposure, which is a key factor in endogenous vitamin D synthesis. The expected number of respondents was 1,600, and the actual number of respondents was 1,827.

The response rate in this study refers to the percentage of individuals who agreed to participate and provided consent out of the total number of individuals invited or contacted. Specifically, the response rate is calculated as the number of participants who completed the survey divided by the total number of individuals invited to participate.

### Social-demographic and health data collection

The field investigation used a structured questionnaire to collect social-demographic and health data. The questionnaire included social-demographic information, dietary data, the outdoor activity time information. The variables used in this study were age group (6–8, 9–11, 12–14, and 15–17 years) and sex, dietary information (daily intake of cereals, vegetables, livestock and poultry meat, fish and shellfish, fruits, legume, eggs and dairy), sleep time on study days, sleep time on rest days, outdoor activity. Each age group may have distinct lifestyle patterns and habits. These differences can influence their dietary habits, sleep patterns, and outdoor activities. Social-demographic and health data were collected through a questionnaire survey and self-reported by the respondents. The questionnaire used in this study is based on the validated China National Nutrition and Health Survey (CNHS), which has been specifically adapted for the Chinese population and has been validated for use in children and adolescents (13).

### Anthropometric measurements

Height was measured without shoes to the nearest 0.2 cm using a portable SECA stadiometer, and weight was measured without shoes and in light clothing to the nearest 0.1 kg on a calibrated beam scale. Body mass index (BMI) was calculated as weight (kg)/height (m^2^). Overweight and obese children were classified according to BMI. The criteria presented in the Chinese National Health Standard WS/T 586–2018 are indeed based on BMI values adjusted for age and sex, and is applicable to both groups (14). While these criteria do not explicitly use growth curves, they provide specific BMI cut-off values for different age and sex groups. These cut-offs serve a similar purpose to BMI/Age indexes and growth curves by allowing for the classification of overweight and obesity based on age-appropriate BMI thresholds.

### Daily intake information collection

To ensure consistency and comparability with established national nutrition surveys, such as the China Nutrition and Health Surveillance (CNHS), we employed a three-consecutive-day dietary recall method. The 24-h dietary recall over the 3 days was conducted by trained investigators. These investigators were specifically trained in dietary assessment techniques to ensure accurate and reliable data collection. They interviewed the participants or their guardians to obtain detailed information about food consumption during the previous 24 h. Among them, the dietary information of children in grades 1–3 of primary school was obtained through their parents/guardians. During home visits over 3 days, dietary data on breakfast, lunch, dinner, and extra meals or snacks were collected. The dietary data collection was conducted using the Chinese Resident Nutrition and Health Survey (CNHS) questionnaire to ensure the quality of dietary recalls.

### Blood sample collection and detection

Blood collection for this study was conducted during the same season to minimize potential seasonal variations in biomarkers. Blood sample were collected to detect the concentration of 25-(OH)D by High-Performance Liquid Chromatography-Tandem Mass Spectrometry (HPLC-MS/MS; AB SCIEX 5500MPX), and we judged deficiency and inadequacy of vitamin D among children and adolescents by boundary values of the concentration of 25-hydroxyvitamin D (15). If the content of serum 25-(OH)D in children and adolescents is <12 μg/L, it is determined as vitamin D deficiency. If the content of serum 25-(OH)D in children and adolescents is <20 μg/L, it is determined as vitamin D inadequacy. Fasting blood glucose were measured by the hexokinase method. According to the Chinese guidelines for type 2 diabetes, Fasting Blood Glucose (FBG) ≥7.0 mmol/L is defined as diabetes, while FBG ≥6.1 and < 7.0 mmol/L is defined as impaired fasting glucose (IFG) (16). We have clarified that in addition to measuring 25-(OH)D levels, we also measured fasting blood glucose and serum retinol levels. According to the Chinese guidelines for Type 2 diabetes, FBG ≥ 7.0 mmol/L is defined as diabetes, while FBG ≥ 6.1 and < 7.0 mmol/L is defined as impaired fasting glucose (IFG). It is important to note that a single high fasting blood glucose level is not sufficient for a definitive diagnosis of type 2 diabetes according to the Chinese guidelines. A diagnosis of type 2 diabetes requires confirmation on a separate day if the patient does not present typical symptoms of diabetes. In our study, we measured random blood glucose levels, but these were not used for clinical diagnosis. Retinol were measured by high performance liquid chromatography. According to the Method for vitamin A deficiency screening (17), we judged marginal deficiency and deficiency of vitamin A among children and adolescents by boundary values of serum retinol level. The serum retinol content of children and adolescents is < 0.2 and < 0.3 ng/ml, it is determined as vitamin A deficiency and marginal deficiency.

### Statistical analysis

The continuous variables were expressed as the mean and standard deviation. The distribution of vitamin D nutritional status among sociodemographic and Clinical status were analyzed by *c*^2^ test. Associations were evaluated using ordinal regression models, with age and sex included in the models to control for their potential influence.

Data processing and statistical analyses were performed using SAS 9.2 software (SAS Institute, Cary, NC, USA). All tests were two-sided, and the level of significance was set at *p* < 0.05.

### Ethical aspects

This study was approved by the Ethics Committee of Zhejiang Provincial Center for Disease Control and Prevention (approval number: 2022-008). The research protocols were carefully explained to the parents/guardians of the school children aged 6–17 years, and all participants and parents/guardians of them provided written informed consent. For children aged 6–17 years, both parental consent and child assent were obtained. All children who participated in the study agreed to have their blood drawn. There were no instances where children opted not to have their blood drawn.

## Results

The study included 1,827 children and adolescents, and the response rate was 97.8%, with 1,760 of them being detected for the concentration of 25-(OH)D. The mean 25-(OH)D concentration was 20.84 ± 6.34 μg/L, and 37.4 and 7.8% children and adolescents aged 6–17 years presented vitamin D insufficiency and deficiency.

The prevalence of vitamin D deficiency varied significantly across different age groups and between male. In the 6–8 age group, 2.7% had vitamin D deficiency. In the 9–11 age group, 4.9% had vitamin D deficiency. In the 12–14 age group, 8.6% had vitamin D deficiency. In the 15–17 age group, 17.55% had vitamin D deficiency. These differences were statistically significant (χ^2^= 152.675, *p* = 0.000). Females had a higher prevalence of vitamin D deficiency (10.8%) compared to males (4.8%), with significant differences (χ^2^= 60.38, *p* = 0.000). There were 8.6, 3.6, and 7.2% presenting vitamin D deficiency in children and adolescents with normal weight, overweight, and obesity, respectively (χ^2^= 10.62, *p* = 0.031). There were 8, 6.3 and 0% presented vitamin D deficiency in children and adolescents with vitamin A status of appropriate, marginal deficiency and deficiency (*c*^2^ = 14.687, *p* = 0.005). Children and adolescents who had 8 h of sleep on study days showed a significantly different serum vitamin D nutritional status compared to those who did not. Specifically, the prevalence of vitamin D deficiency was lower among those who slept 8 h on study days compared to those who did not (χ^2^= 34.69, *p* = 0.000). There was a significant difference in serum vitamin D nutritional status between children and adolescents who had 2 h of outdoor activity per day and those who did not (χ^2^= 7.31, *p* = 0.026). A significant difference was observed in serum vitamin D nutritional status between children and adolescents who consumed 145 g of livestock and poultry meat per day and those who did not. Specifically, the prevalence of vitamin D deficiency was higher among those who consumed 145 g of livestock and poultry meat per day compared to those who did not (χ^2^= 6.716, *p* = 0.035; Table 1).

**Table 1:** The characteristics of sociodemographic and clinical status among children and adolescent with appropriate, insufficiency and deficiency of vitamin D.

**Sociodemographic and clinical parameters**	**Vitamin D serum concentration (**μ**g/L)**			***c* ^2^**	***p*-value**
	**25(OH)D ≥20**	**25(OH) < 20 and 25(OH) ≥12**	**25(OH) < 12**		
**Age group**					
6–8 years	13 (71.4%)	127 (26%)	13 (2.7%)	152.675	0.000
9–11 years	283 (59.8%)	167 (35.3%)	283 (4.9%)		
12–14 years	193 (44.7%)	202 (46.8%)	37 (8.6%)		
15–17 years	139 (38%)	163 (44.5%)	64 (17.5%)		
**Sex**					
Girls	401 (45.9%)	378 (43.3%)	94 (10.8%)	60.38	0.000
Boys	563 (63.5%)	281 (31.7%)	43 (4.8%)		
**Overweight/obesity**					
Normal weight	717 (53.9%)	499 (37.5%)	115 (8.6%)	10.62	0.031
Overweight	151 (60.9%)	88 (35.5%)	9 (3.6%)		
Obesity	96 (53%)	72 (39.8%)	13 (7.2%)		
**Glucose screening**					
Fasting blood glucose ≥7.0 mmol/L or HbA1c ≥6.5%	2 (28.6%)	4 (57.1%)	1 (14.3%)	4.716	0.095
Fasting blood glucose < 7.0 mmol/L or HbA1c < 6.5%	58 (69%)	21 (25%)	5 (6%)		
**Vitamin A status**					
Appropriate	848 (53.4%)	612 (38.6%)	127 (8%)	14.687	0.005
Marginal deficiency	108 (67.9%)	41 (25.8%)	10 (6.3%)		
Deficiency	3 (42.9%)	4 (57.1%)	0 (0%)		
**Sleep time on study days**					
≥8 h	880 (56.4%)	577 (37%)	102 (6.5%)	34.69	0.000
< 8 h	84 (41.8%)	82 (40.8%)	35 (17.4%)		
**Sleep time on rest days**					
≥8 h	906 (55%)	614 (37.3%)	126 (7.7%)	0.986	0.611
< 8 h	58 (50.9%)	45 (39.5%)	11 (9.6%)		
**Outdoor activity**					
≥2 h	699 (55.4%)	453 (35.9%)	109 (8.6%)	7.319	0.026
< 2 h	265 (53.1%)	206 (41.3%)	28 (5.6%)		
**Intake of livestock and poultry meat**					
< 145 g/d	330 (57.8%)	211 (37%)	30 (5.3%)	6.716	0.035
≥145 g/d	106 (56.1%)	63 (33.3%)	20 (10.6%)		
**Intake of eggs**					
< 57 g/d	273 (55.8%)	184 (37.6%)	32 (6.5%)	3.534	0.171
≥57 g/d	101 (64.3%)	48 (30.6%)	8 (5.1%)		

In the ordinal regression model (χ^2^= 26.572, *p* = 0.002), it is observed age group (Wald *c*^2^ = 5.921, OR = 0.475, *p* = 0.015), sex (Wald χ^2^= 6.206, OR = 0.192, *p* = 0.013), overweight/obesity (Wald χ^2^= 3.894, OR = 4.967, *p* = 0.048), and outdoor activity time (Wald χ^2^= 4.113, OR = 0.259, *p* = 0.043) were the influencing factors of children and adolescents with insufficiency/deficiency vitamin D status (Table 2).

**Table 2:** Ordinal regression of influencing factors of children and adolescent with appropriate, insufficiency and deficiency.

**Variables**	**Classification**	**β**	**Wald *c*^2^**	**OR**	***p*-value**
Age group	6–8, 9–11, 12–14, 15–17 years	−0.743	5.921	0.475	0.015
Sex	Boys, girls	−1.648	6.206	0.192	0.013
BMI	Normal weight, overweight, obesity	1.603	3.894	4.967	0.048
Glucose screening	Fasting blood glucose ≥7.0 mmol/L or HbA1c ≥6.5%, fasting blood glucose < 7.0 mmol/L or HbA1c < 6.5%	−2.390	2.119	0.094	0.145
Vitamin A status	Appropriate, marginal deficiency, deficiency	0.715	0.512	2.044	0.474
Sleep time on study days	≥8, < 8 h	0.603	0.537	1.828	0.463
Outdoor activity time	≥2, < 2 h	−1.346	4.113	0.259	0.043
Intake of livestock and poultry meat (%)	< 145, ≥145 g/d	−0.304	0.209	0.738	0.648
Intake of eggs (%)	< 57, ≥57 g/d	−0.474	0.450	0.621	0.502

## Discussion

Our study revealed a significant prevalence of vitamin D insufficiency and deficiency among children and adolescents in Zhejiang province, China. The main influencing factors identified were age group, sex, overweight/obesity, and outdoor activity time.

Micronutrient malnutrition or “hidden hunger” is a global health problem. We acknowledge that iron deficiency is the most common form of anemia and ~25% of people worldwide have anemia (18). However, vitamin D deficiency is also a serious public health problem, affecting nearly 50% of the global population (8, 9, 19, 20). Our findings indicate that the prevalence of vitamin D deficiency in our study population is similar to or even higher than that reported in many other regions globally. For example, a study in the United States found that about 35% of adults are vitamin D deficient, while over 80% of adults in Pakistan, India, and Bangladesh are vitamin D deficient (18). Vitamin D is an elemental fat soluble vitamin that works a significant role in skeletal system and other organs and tissues (21). A study carried out in 2012 among children aged under 18 years in China found the prevalence of vitamin D insufficiency or deficiency was 23.3% (22). A study among adolescents in Brazil found vitamin D deficiency and insufficiency was 41.5 and 25.8%, respectively (23). In this provincial representative survey in the eastern coastal areas of China, the prevalence of vitamin D insufficiency and deficiency among children and adolescents was high (37.4 and 7.8%). Therefore, the nutritional status of vitamin D should be attracted public attention as a public health issue.

Vitamin D deficiency and insufficiency may result from inadequate nutritional vitamin D intake, inadequate sunlight exposure, disorders reducing vitamin D absorption, and conditions that impair vitamin D conversion into active metabolites (24, 25). It is important to note that while 1,25-dihydroxyvitamin D (1,25-(OH)2D) is the biologically active form of vitamin D, 25-(OH)D levels are used to define vitamin D status because they provide a more stable and reliable measure of overall vitamin D status. Adolescence is a pivotal point in the life course, characterized by transformative physical, cognitive, and emotional growth, an openness to change, and a drive to reshape the social environment. It offers unique opportunities to adopt changes in diet and physical activity that can persist into later life (26). Previous study suggested the prevalence of vitamin D deficiency increased with age. A study in healthy children found negative correlation between vitamin D level and age (27). Another study showed that vitamin D level decreased by 2.164 nmol/L for each year of age (28). Consistent with these literature, in this study, there were 2.7, 4.9, 8.6, and 17.55% children and adolescents presented vitamin D deficiency in 6–8, 7–9, 12–14, and 15–17 age group. Adolescents are particularly at risk for vitamin D deficiency due to several factors. First, adolescents often have less sun exposure than younger children. This can be attributed to lifestyle changes, such as increased time spent indoors for school and extracurricular activities, as well as cultural practices that may limit outdoor exposure. Second, the increased demand for vitamin D during puberty may also play a role. Adolescents experience rapid growth and development, which increases their need for vitamin D to support bone health and other physiological functions. Finally, adolescents are less likely to take vitamin D supplements compared to younger children, which can further exacerbate the problem. These findings underscore the importance of targeting older children and adolescents to prevent vitamin D deficiency. Early intervention could potentially reduce the prevalence of deficiency in adolescence. In other words, while it is crucial to focus on prevention strategies for older children and adolescents, we must not overlook the importance of addressing vitamin D deficiency in younger children as well. In our previous study in 2016–2017, significant difference was observed on the concentration of 25-hydroxyvitamin D (25(OH)D) both between males and females (28). Consistent with these literature, in this study, there were 10.8 and 4.8% children presented vitamin D deficiency in females and males with significant difference. These sex differences in vitamin D status can be attributed to various factors, including differences in lifestyle, sun exposure, and physiological characteristics. Sunlight exposure is an important source of vitamin D. Compared to boys, girls have less outdoor activities, and at the same time, girls have an earlier puberty and a higher demand for vitamin D, which may be the reason for sex differences in vitamin D. Child and adolescent overweight/obesity has been demonstrated to be partially associated with vitamin D deficiency (29, 30). Notably, obesity can lead to lower circulating 25-(OH)D levels due to sequestration in adipose tissue (31, 32). While in this study, there were 8.6, 3.6, and 7.2% presented vitamin D deficiency in children and adolescents with normal weight, overweight and obesity. We also conducted an Analysis of Variance (ANOVA) analysis with age and sex as covariates to adjust for their potential confounding effects on the relationship between BMI and insufficiency/deficiency vitamin D status. However, we did not find significant differences after adjusting for these covariates. This suggests that the relationship between BMI and vitamin D deficiency is more complex than initially thought. While BMI is a recognized risk factor for insufficiency/deficiency vitamin D status, the influence of age and sex may be substantial enough to modify the observed associations. Outdoor activities increase body vitamin D levels through sun-induced synthesis of vitamin D (33). Abboud et al. (34) reported that specific uptake of 25(OH)D into muscle may contribute to the long half-life of 25(OH)D in blood, and exercise may extend the half-life of serum 25(OH)D. A study in China reported that longer time of outdoor activity were positively linked to higher serum 25(OH)D3 (35). Consistent with these literature, in this study, there were significant difference of children and adolescents with or without outdoor activity time of 2 h. To further explore the factors influencing insufficiency/deficiency vitamin D status, we performed ordinal regression analyses. We included variables that showed significant differences in the univariate analysis, along with age and sex. In the ordinal regression model, when adjusted by age and sex factors, outdoor activity time and overweight/obesity were the influencing factors of children and adolescents with insufficiency/deficiency vitamin D status. This founding also suggested that sedentary habits could reduce time spent outdoor in sunlight, and increase the risk of obesity. In order to prevent vitamin D insufficiency/deficiency, there are a few measures we can take. Basically, it is necessary correct the risk factors by means of increasing outdoor activities and controlling weight.

Our study has several limitations that should be considered when interpreting the results. First, our questionnaire did not include specific details about the amount of skin exposed to sunlight or the typical clothing worn during outdoor activities. This lack of information limits our ability to accurately estimate the amount of skin exposed to sunlight, which is a key factor in endogenous vitamin D synthesis. Future studies should consider including questions about typical clothing and skin exposure to provide a more comprehensive assessment of sunlight exposure. Second, while we conducted the study during the summer months to minimize seasonal variations in vitamin D levels, we did not account for individual variations in sun exposure due to factors such as geographic location, weather conditions, and personal habits. Third, our study relied on self-reported data for outdoor activity time, which may introduce recall bias. Additionally, we did not consider factors such as physical activity intensity and sun protection measures. Fourth, our questionnaire included questions about the use of vitamin D and calcium supplements, but due to a high number of missing values and the low prevalence of supplement use among the participants, we were unable to perform a meaningful analysis of the data. This limitation should be considered when interpreting the results related to potential supplementation practices among the study population. Future studies should consider including more detailed questions about supplement use and ensuring higher response rates to provide a more comprehensive assessment of supplementation practices. Finally, the cross-sectional design of our study limits our ability to establish causality between the identified factors and vitamin D status. Nevertheless, our study has several strengths, especially it is a provincial representative survey on children and adolescents in the eastern coastal areas of China. Moreover, physical examination and dietary intake as well as outdoor activity time were included to explore vitamin D status and influencing factors.

## Conclusions

Our study assessed the nutritional status of vitamin D and analyzed the influencing factors of vitamin D deficiency among 1,827 children and adolescents in the eastern coastal areas of China. The results indicated a significant prevalence of vitamin D insufficiency and deficiency. Key influencing factors included age group, sex, overweight/obesity, and outdoor activity time. These findings highlight the need for targeted interventions to improve vitamin D levels in children and adolescents. Specifically, promoting adequate outdoor activity and addressing overweight/obesity could be effective strategies to enhance vitamin D status in this population.

## Data Availability

The raw data supporting the conclusions of this article will be made available by the authors, without undue reservation.
